# RXNMapper-v2: updated validation and evaluation on expanded chemical space

**DOI:** 10.1039/d6dd00425c

**Published:** 2026-10-13

**Authors:** Yves Grandjean, Sacha Raffaud, Annie M. Westerlund, Philippe Schwaller, Samuel Genheden, Jean-Louis Reymond

**Affiliations:** a Department of Chemistry, Biochemistry and Pharmaceutical Sciences, University of Bern Freiestrasse 3 3012 Bern Switzerland; b Laboratory of Artificial Chemical Intelligence (LIAC), Institute of Chemical Sciences and Engineering, Ecole Polytechnique Fédérale de Lausanne (EPFL) 1015 Lausanne Switzerland; c Molecular AI, Discovery Sciences, R & D, AstraZeneca Gothenburg SE-431 83 Mölndal Sweden jean-louis.reymond@unibe.ch

## Abstract

Atom-to-atom mapping (AAM) assigns reactant atoms to product atoms and is fundamental for reaction classification, template extraction, and synthesis planning. RXNMapper derives AAM from the attention weights of a transformer model trained by masked language modeling on unmapped reaction SMILES. However, its original model selection relied on USPTO-50K, a validation set later shown to contain erroneous mappings. Here we revisit RXNMapper using a curated validation set of 4′533 reactions from the dataset Pistachio. We retrained ALBERT-based RXNMapper models on USPTO-full and Pistachio and selected the best head, layer, and checkpoint on this new validation set. To improve the comparison of AAM algorithms, we developed an evaluation framework that removes ambiguous reference reactions and compares predictions based on reaction graph isomorphism rather than direct string matching, avoiding false negatives from inconsistent formats and symmetry-equivalent mappings. Across the golden dataset, Pistachio, and an internal AstraZeneca medicinal chemistry benchmark, the updated models outperform the original RXNMapper and LocalMapper, while rearrangements remain persistent challenges.

## Abstract 2

Atom-to-atom mapping (AAM) assigns reactant atoms to product atoms and is fundamental for reaction classification, template extraction, and synthesis planning.

## Graphical abstract

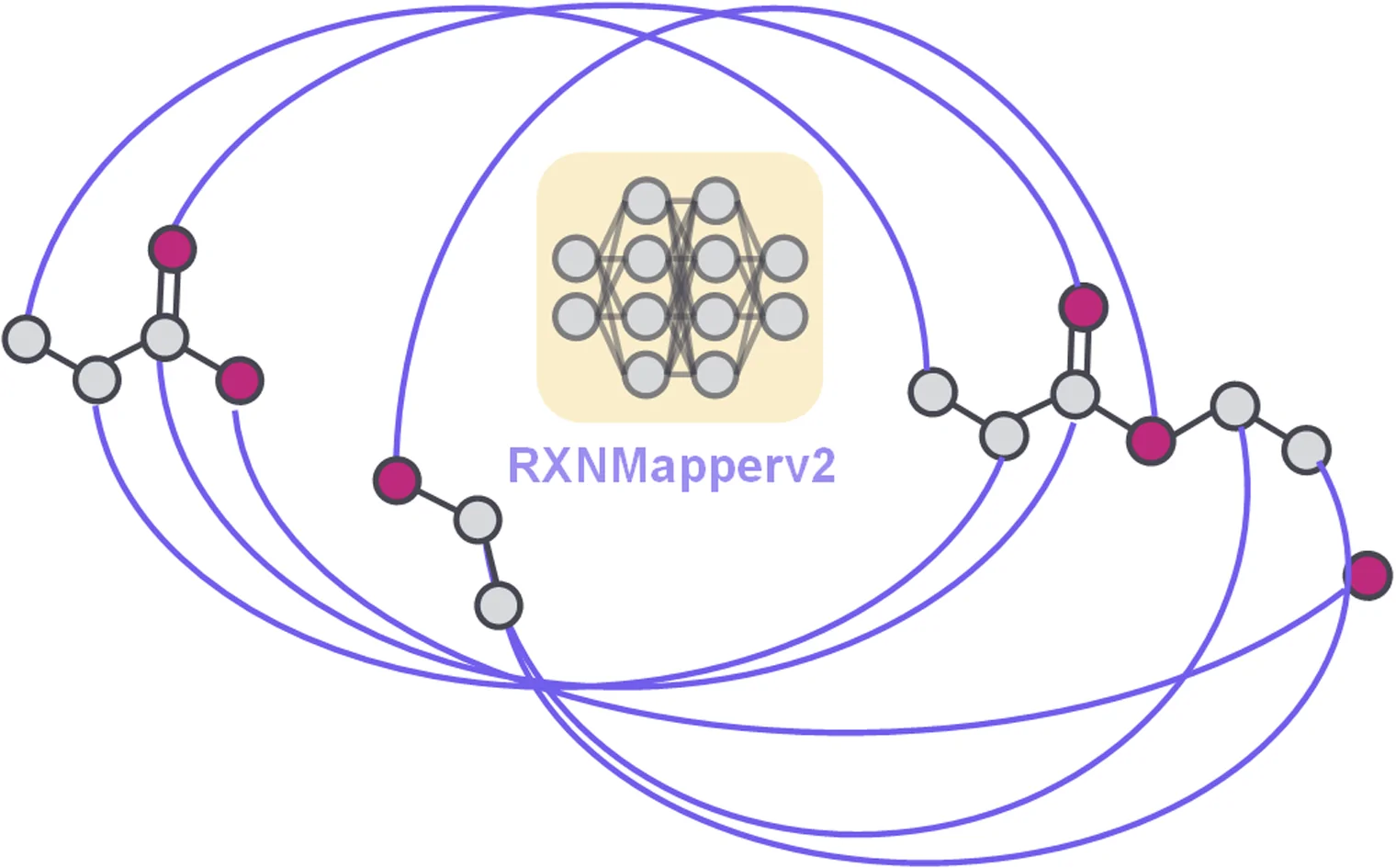

## Introduction

Atom-to-atom mapping (AAM), namely the assignment of reactant atoms to their corresponding product atoms, is a basic operation in cheminformatics as it describes how atoms are redistributed during a reaction.^1^ Accuracy of atom mapping has implications on downstream tasks such as reaction search,^1^ reaction classification^2,3^ reaction template extraction for computer-assisted synthesis planning,^4–13^ and several template-free applications including reaction-center tagging,^14–19^ reaction tokenization,^20^ and reaction representation learning for property prediction.^21–24^

Early AAM methods relied on structural comparison, optimization procedures, or expert-defined transformation rules to assign atom correspondences.^1,25–40^ More recently, neural-network approaches based on transformers^41–43^ and graph models have enabled data-driven atom mapping, such as RXNMapper,^44^ Graphormer,^45^ LocalMapper,^46^ and AMNet.^47^ Among these, RXNMapper is particularly attractive because it is trained by masked language modelling (MLM) on unmapped reaction SMILES, making it simple to train and fast to apply.^44^ However, its original model selection relied on a mapped validation set derived from USPTO-50K, which was later shown to contain erroneous mappings, while LocalMapper was subsequently reported to outperform RXNMapper.^46^

Here we revisit the RXNMapper approach using a curated validation set designed to cover the chemical space of Pistachio. We first establish an evaluation framework for atom mapping that removes ambiguous reference reactions and compares predictions based on reaction graph isomorphism rather than direct string matching. We then retrain ALBERT-based RXNMapper models on USPTO-full (2.8 M reactions, RXNMapper-v2) and Pistachio (15.06 M reactions, RXNMapper-P) and improve model selection at the level of head, layer, and checkpoint using the curated validation set from Pistachio (4′533 reactions). The original RXNMapper, the two updated models, and LocalMapper are compared on three benchmarks: the golden dataset (1′758 reactions),^46,48^ a Pistachio test set (16′248 reactions), and an internal AstraZeneca medicinal chemistry dataset (108′611 reactions). We find that reliable model selection has a larger effect on performance than retraining on a new reaction corpus, and that the updated RXNMapper models outperform both the original RXNMapper and LocalMapper across the datasets examined here. To support reproducibility, we provide the training scripts, model-selection workflow, updated checkpoints, and evaluation framework in dedicated repositories.

## Methods

### Datasets

We used four reaction datasets in this study. USPTO-full, composed of 2′847′312 reactions extracted from the United States Patent and Trademark Office by Lowe,^49,50^ contains 2.8 M unmapped reaction SMILES and was used to train the original RXNMapper and the updated RXNMapper-v2 models. USPTO-50K, introduced by Schneider *et al.*,^3^ consists of 50 K reactions sampled from USPTO-full and atom-mapped and classified with NameRXN (v2.1.84). The golden dataset, introduced by Lin *et al.*^48^ and later filtered by Chen *et al.*^46^ contains 1′758 reactions and was used as a benchmark for atom-mapping performance. Pistachio,^51^ the proprietary reaction dataset from NextMove, contains 15′065′981 mapped and classified reactions in the 2025Q2 release. To obtain a benchmark covering broad chemical space, we sampled up to 10 reactions per reaction class randomly across the 2′460 available classes and performed a random stratified 20/80 split of the resulting set by reaction class into validation (4′533 reactions) and test (16′248 reactions) subsets. Finally, an internal AstraZeneca medicinal chemistry dataset containing 108′611 mapped (by NextMove) reactions recorded between January 2024 and June 2025 with yields above 20% was used as a third benchmark representing a narrower chemical space.

### Models, training and inference

We compared four atom-mapping models: the original RXNMapper,^44^ LocalMapper,^46^ and two updated ALBERT-based RXNMapper models retrained in this work. The original RXNMapper was trained by Masked Language Modeling (MLM) on USPTO-full and used as published. LocalMapper was used from the author's public implementation. The two updated RXNMapper models, RXNMapper-v2 and RXNMapper-P, were trained with a new MLM training pipeline on the unlabeled (*i.e.* not atom-mapped) USPTO-full (2′847′312 reactions) and Pistachio (15′065′981 reactions), respectively, using the same ALBERT architecture as the original model and training each model for 1.5 M steps. For RXNMapper-v2, the original vocabulary was used, whereas a new one was built for RXNMapper-P. Vocabularies were used to tokenize the reactions before running MLM. For each model, the final atom-mapping configuration was selected by choosing the best head, layer, checkpoint combination on the Pistachio validation set. All heads of the three last layers (indices 9, 10, 11) were used to atom-map the validation set reactions across all 150 model training checkpoints. Our evaluation pipeline was used to find the head/layer/checkpoint that led to the best performance on the validation set.

### Standardization and evaluation of atom-mapped reactions

Before evaluation, atom-map indices were canonicalized using a deterministic ordering procedure derived from the original RXNMapper workflow. Ground-truth reactions were then screened for formatting inconsistencies and excluded when they contained duplicated product-side atom-map indices, product atoms without reactant-side counterparts, invalid or non-canonicalizable structures, unsanitizable molecules, or unmapped product atoms. Excluded reactions were flagged to allow later inspection.

For evaluation, each atom-mapped reaction was converted into a labeled reaction graph. Atom correspondences between reactant and product sides were encoded through shared atom-map indices, and graph equivalence between ground-truth and predicted reactions was assessed by labeled graph isomorphism. This comparison was implemented with the VF2 isomorphism algorithm as available in NetworkX.^52,53^ Graph-based comparison treats symmetry-related but chemically equivalent atom mappings as identical.

### TMAPs

TMAP^54^ was used to visualize the structure of the reaction error sets. Reactions were represented with RXNFP fingerprints^55^ derived from reaction SMILES, and nearest-neighbor relationships were computed using cosine distance. The resulting maps were used to examine how incorrectly predicted reactions are distributed in reaction space and whether distinct regions of model agreement or failure could be identified.

## Results and discussion

### Validation set design

In the benchmarking study used for LocalMapper (a graph neural network trained *via* human-in-the-loop currently state-of-the-art on USPTO-50 K),^46^ the authors reported errors in the USPTO-50 K dataset,^3^ including in the validation and test sets, and removed part of these automatically, leaving 969 of the original 1000 validation reactions. Because USPTO-50 K is widely used for benchmarking atom-mapping models, we examined reactions where AAM from RXNMapper and LocalMapper differed, and discovered around 100 additional reactions which were clearly unreliable as atom-mapping references. We observed typographical errors in reactions (Fig. 1a), errors in the ground-truth atom-maps (Fig. 1b), and cases in which multiple atom mappings could reasonably be assigned (Fig. 1c). We also found reactions whose recorded transformation appeared chemically questionable. Taken together, these issues make USPTO-50 K poorly suited as a validation or test set for the present study.

**Fig. 1:**
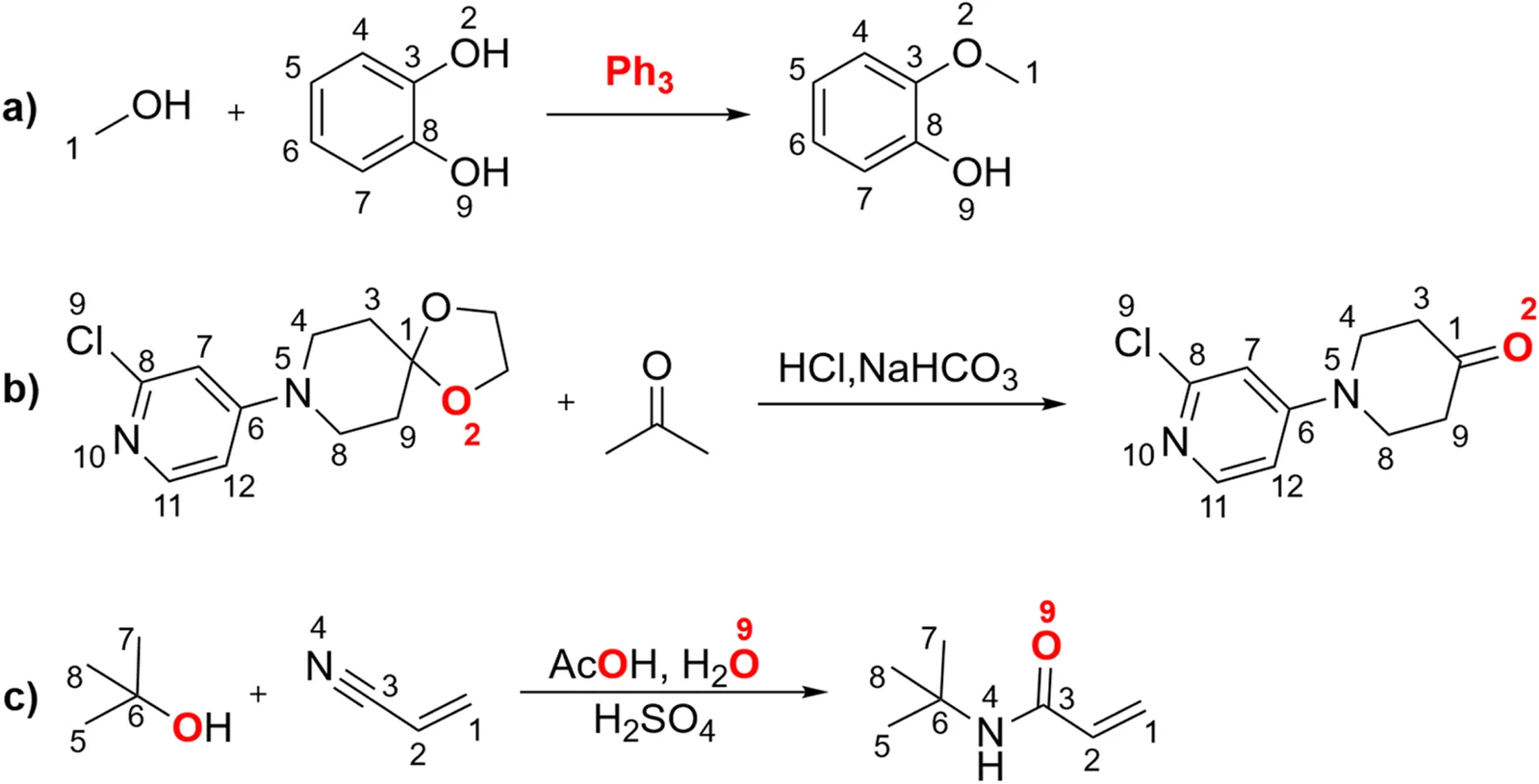
Examples of NameRXN 2.1.84 atom-mapping errors or ambiguities in USPTO-50 K. (a) Typographical and reagent-description errors in a Mitsunobu reaction (Ph_3_ instead of the full reagent set PPh_3_/DEAD). (b) Incorrect atom mapping in an unbalanced reaction (the product O-atom should correspond to a water molecule or the acetone O-atom). (c) A reaction for which several atom mappings are chemically plausible, which can lead to false negatives during evaluation (the product amide O-atom might correspond to any of the three O-atoms in reagents and reactants).

We therefore used Pistachio (2025Q2) as the reference dataset for model selection. Pistachio covers broader chemical space and provides NameRXN-based reaction classes in a three-level *x*.*y*.*z* scheme, where *x* denotes the superclass and *y*.*z* the reaction class. To obtain representative validation and test sets, we sampled up to 10 reactions per reaction class and applied a stratified split by reaction class, assigning up to 8 reactions per class to the test set and up to 2 to the validation set. This yielded 16′248 test reactions and 4′533 validation reactions. The Pistachio validation set was then used to select the best model configuration in the remainder of this work.

### Training RXNMapper models

Because the original RXNMapper publication released model checkpoints but not the corresponding training scripts, we implemented a new training pipeline reproducing the original ALBERT setup. Two models were trained by unsupervised learning on unmapped reaction SMILES: one on USPTO-full (RXNMapper-v2) and one on the full Pistachio dataset (RXNMapper-P) (Fig. 2a). The Pistachio-trained model was included to test whether exposure to a broader chemical space improves atom-mapping performance under the same unsupervised training scheme.

**Fig. 2:**
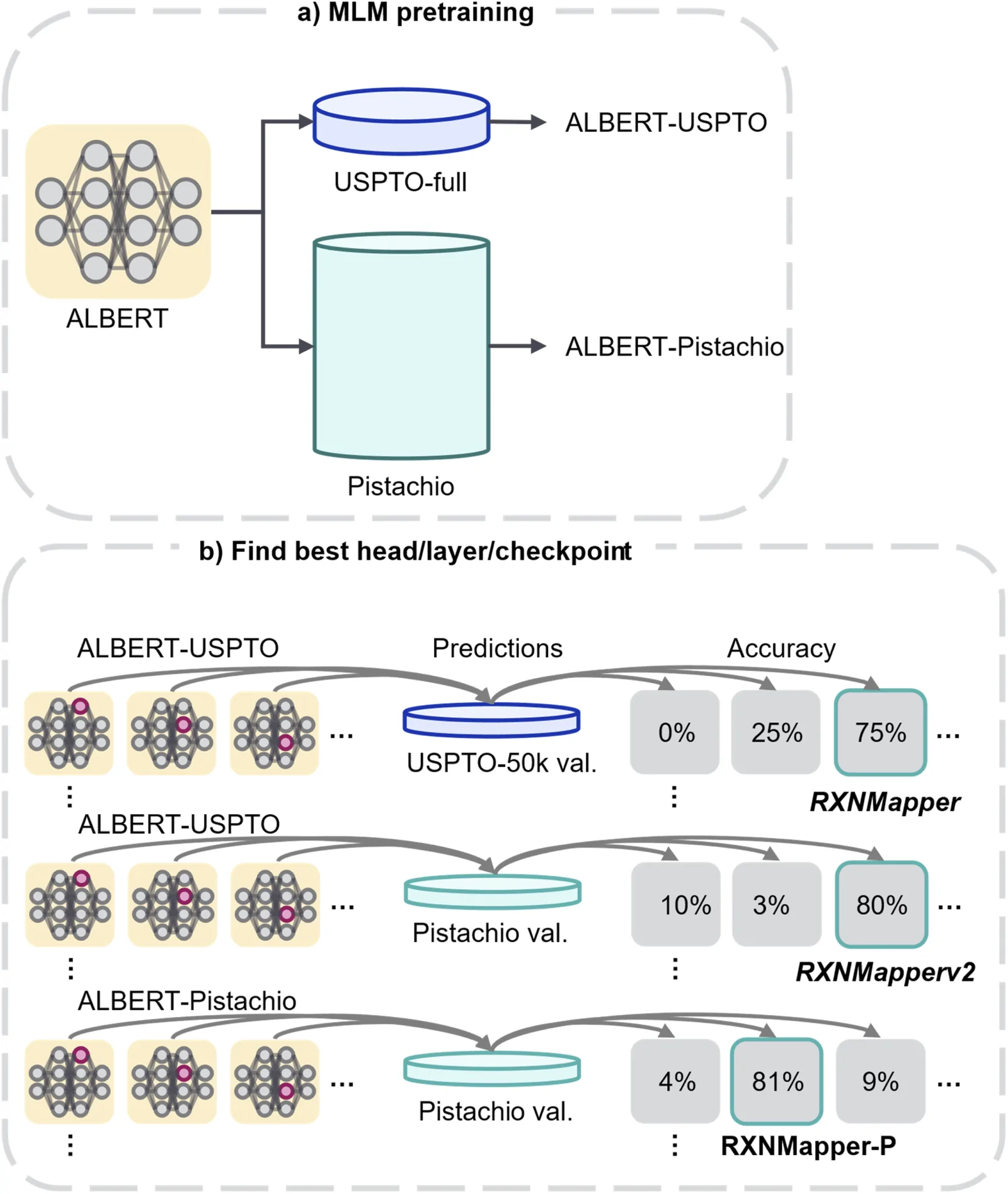
Overview of RXNMapper training and model selection. (a) ALBERT pretraining by masked language modeling on large corpora of unmapped reaction SMILES. (b) Selection of the best head/layer/checkpoint combination by performance comparison on the validation set.

After pretraining, the atom-mapping head was selected empirically. In the RXNMapper framework, atom mapping is derived from the attention pattern of a single head, which is used to associate each product atom with the corresponding reactant atom. We therefore evaluated head, layer, and checkpoint combinations on the Pistachio validation set and retained the best-performing configuration as the final model (Fig. 2b).

### Preprocessing and evaluation of atom-mapping predictions

Atom-mapped reactions are not represented consistently across datasets, which can introduce false negatives during evaluation. To address this, we developed a preprocessing and evaluation pipeline extending the approach of Schwaller *et al.*^44^ After atom-map canonicalization, ground-truth reactions were excluded when (1) two or more product atoms shared the same mapping number, (2) a product atom had no mapped counterpart on the reactant side, or (3) a product atom was left unmapped (Fig. 3a). These cases were removed because they make the reference mapping ambiguous and unsuitable for benchmarking.

**Fig. 3:**
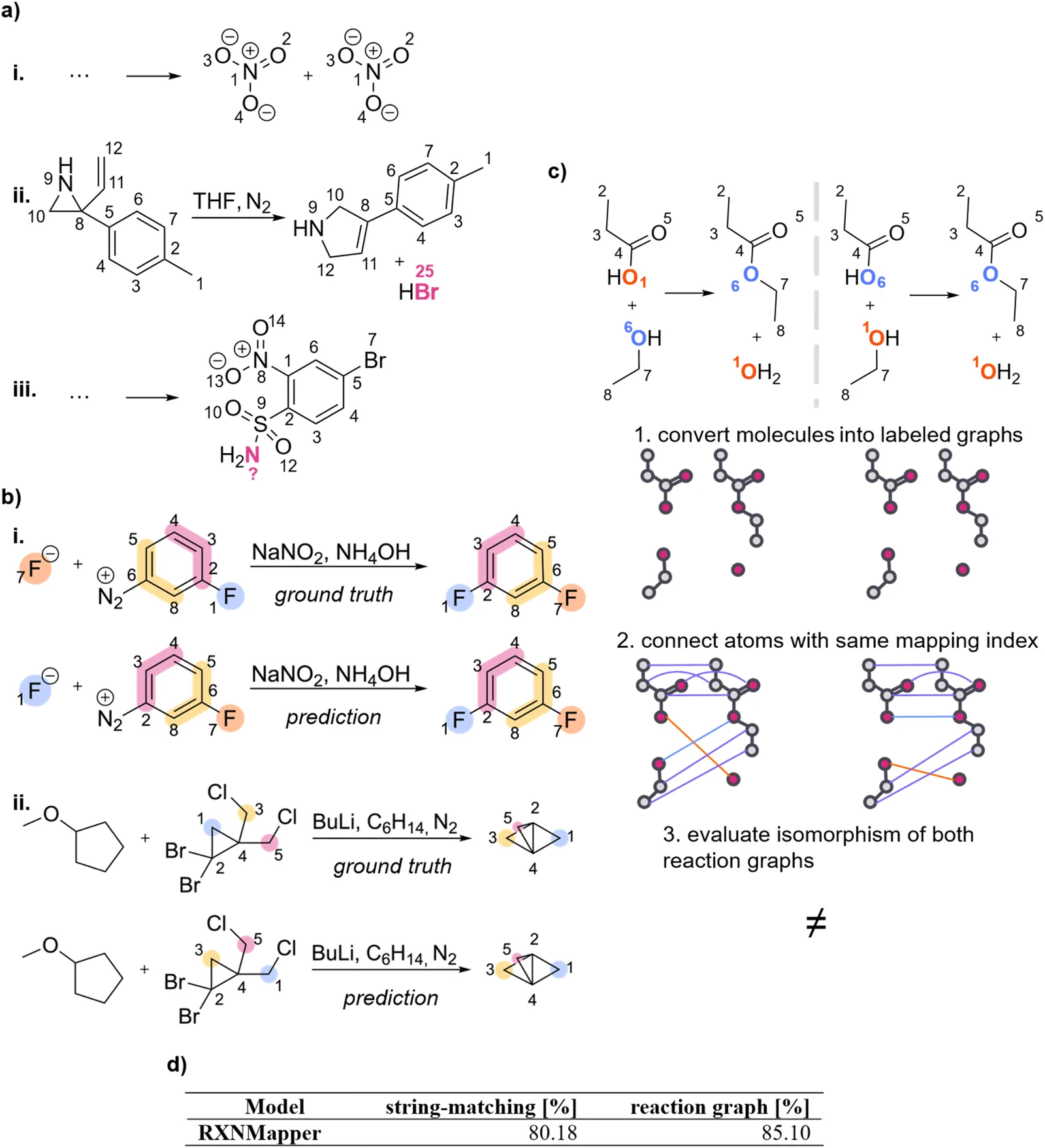
Preprocessing and evaluation framework. (a) Atom-mapped reaction formats excluded during preprocessing: (i) duplicated product-side atom-map indices, (ii) product-side atom-map indices without reactant-side counterparts, and (iii) unmapped product atoms. (b) Examples of highly symmetrical reaction pairs that require symmetry-aware evaluation. (c) Graph-based comparison procedure in which atom-mapped reactions are converted into labeled reaction graphs and tested for isomorphism. (d) Difference in measured performance between string matching and reaction graph comparison for the original RXNMapper model on the Pistachio test set (13′862 reactions).

We then replaced string-based comparison with graph-based evaluation. For each ground-truth/predicted pair, the mapped reactions were converted into reaction graphs and compared by labeled graph isomorphism (Fig. 3c, see Theory section in the SI). Under this representation, two mappings were considered equivalent when they described the same underlying transformation, including symmetry-related alternatives (Fig. 3b). This avoids penalizing chemically correct predictions that differ only by an alternative but equivalent atom numbering.

On the Pistachio test set, the measured accuracy of the original RXNMapper differed by 4.92 percentage points depending on whether evaluation was based on string matching or reaction graph comparison (Fig. 3d). This gap arises from false negatives produced by the string-based procedure, especially for highly symmetrical reactions (Fig. 3b). These results show that robust atom-mapping evaluation requires both filtering of ambiguous references as well as symmetry-aware comparison of predicted and reference mappings. Reactions removed from the evaluation are not necessarily wrong, but their atom-mapping format should be updated to remove all ambiguity and allow a clear evaluation.

### Performance of the updated model on different chemical spaces

We next compared the two updated RXNMapper models with the original RXNMapper and with LocalMapper. The models were benchmarked on three datasets representing complementary reaction spaces: the golden dataset as an open benchmark, the Pistachio test set as a broad chemical space benchmark, and the AstraZeneca medicinal chemistry set as a narrower pharmaceutical benchmark. In all cases, ground-truth and predicted reactions were preprocessed before evaluation. This left 1′758 reactions in the golden dataset, 13′862 in the Pistachio test set, and 103′286 in the AstraZeneca set.

On the preprocessed golden dataset, RXNMapper reached 86.42% accuracy, RXNMapper-v2 89.12%, RXNMapper-P 88.23%, and LocalMapper 89.02% (Table 1). On the preprocessed Pistachio test set, RXNMapper reached 85.10%, RXNMapper-v2 87.25%, RXNMapper-P 87.96%, and LocalMapper 80.59%. On the preprocessed AstraZeneca medicinal chemistry set, the same ranking was observed, with accuracies of 96.79% for RXNMapper, 97.34% for RXNMapper-v2, 97.35% for RXNMapper-P, and 94.13% for LocalMapper.

**Table 1:** Evaluation by reaction graph comparison of the different atom-mapping models on the preprocessed golden dataset (1′758 reactions), Pistachio test set (13′862 reactions), and internal AstraZeneca medicinal chemistry set (103′286 reactions)

Model	Golden dataset [%]	Pistachio test set [%]	AZ med chem set [%]
RXNMapper	86.42	85.10	96.79
RXNMapper-v2	**89.12**	87.25	97.34
RXNMapper-P	88.23	**87.96**	**97.35**
LocalMapper	89.02	80.59	94.13

Across the three benchmarks, the original RXNMapper consistently gives the lowest accuracy among the ALBERT-based models. The largest gain is observed when the original USPTO-trained model is retrained and reselected under the updated workflow, while additional pretraining on Pistachio provides only a smaller benefit. On Pistachio, RXNMapper-v2 improves by 2.15 percentage points over the original model, whereas RXNMapper-P adds a further 0.71 points. On the AstraZeneca set, RXNMapper-v2 and RXNMapper-P are nearly identical (97.34% and 97.35%), indicating that a representative validation set has a larger effect than retraining on a broader reaction corpus.

The absolute accuracy values also show a clear benchmark effect. Performance is lowest on Pistachio, where even the best model remains below 88%, and highest on the AstraZeneca medicinal chemistry set, where all models exceed 94%. This is consistent with Pistachio covering a broader and more difficult reaction space, whereas the AstraZeneca benchmark reflects a narrower medicinal chemistry domain.

LocalMapper performs competitively on the golden dataset but underperforms on Pistachio and AstraZeneca relative to the ALBERT-based models. This trend may reflect differences in the training domain, as LocalMapper was developed on USPTO-centered data, whereas Pistachio spans a broader chemical space.

### Reaction class performance

We used the NameRXN superclass annotation of the Pistachio test set to examine whether model performance differs across broad reaction classes (TMAP of the test set colored by NameRXN superclass, Fig. S1). Table 2 reports the number of mistakes per superclass for each model. Across the 12 superclasses, LocalMapper gives the highest error count overall. Among the ALBERT-based models, RXNMapper-P gives the lowest number of errors in 9 of the 12 superclasses, including the frequent superclasses 4 (heterocycle formation) and 9 (functional group interconversion, FGI), whereas RXNMapper-v2 gives the lowest counts in superclasses 3 (C–C bond formation), 5 (protections), and 7 (reductions). Relative to superclass size, superclass 12 shows 5/5 reactions in the error set, but this category contains only five miscellaneous examples. Among the larger classes, the highest error-set proportions are observed for superclass 4 (heterocycle formation) and superclass 8 (oxidations), both at 36.7%, followed by superclass 10 (functional group addition, 33.6%), superclass 9 (functional group interconversion, 29.6%), and superclass 3 (C–C bond formation, 27.8%). In absolute terms, superclasses 4 (heterocycle formation), 9 (FGI), and 3 (C–C bond formation) contribute the largest numbers of reactions to the error set, with 916, 829, and 758 reactions, respectively (Table 2).

**Table 2:** Number of mistakes per superclass for each model on the Pistachio test set (13′862 reactions). The error-set column gives the number of reactions mistaken by at least one model and the corresponding percentage within that superclass

Class description	Super class	RXNMapper	RXNMapper -v2	RXNMapper -P	LocalMapper	Error set [# (%)]	Full test set
Heteroatom alkylation/arylation	1	187	173	**166**	274	386 (19.6)	1′974
Acylation related processes	2	185	176	**152**	198	298 (23.5)	1′269
C–C bond formation	3	449	**360**	371	576	758 (27.8)	2′727
Heterocycle formation	4	486	425	**394**	657	916 (36.7)	2′494
Protections	5	11	**2**	3	28	28 (23.3)	120
Deprotections	6	29	17	**12**	83	107 (14.4)	741
Reductions	7	39	**37**	45	68	102 (15.0)	679
Oxidations	8	127	112	**96**	153	221 (36.7)	603
Functional group interconversion	9	478	399	**375**	558	829 (29.6)	2′804
Functional group addition	10	58	39	**37**	65	93 (33.6)	277
Separation processes	11	13	22	**14**	26	42 (24.9)	169
Miscellaneous	12	**4**	5	**4**	5	5 (100)	5
Total		2′066	1′767	1′669	2′691	3′785 (27.3)	13′862

To further analyze model behavior, we examined where models make mistakes and how errors overlap across them. The error set comprises all reactions that have been wrongly predicted by at least one of the models. Table 3 summarizes the 3′785 reactions in the Pistachio error set. Exactly one model is wrong for 1′640 reactions, exactly two models for 706 reactions, exactly three models for 615 reactions, and all four models for 824 reactions. Among single-model errors, LocalMapper accounts for 1′097 reactions, compared with 277 for the original RXNMapper, 139 for RXNMapper-v2, and 127 for RXNMapper-P.

**Table 3:** Number of mistaken models on the Pistachio test set. Number of times each model belongs to a given error-overlap class

Mistaken models	ALBERT orig.	ALBERT repr.	ALBERT pistachio	LocalMapper	Error set
1	284	139	**127**	1′090	1′640
2	433	291	**279**	409	706
3	525	513	439	**368**	615
4	824	824	824	824	824
Total	2′066	1′767	**1′669**	2′691	3′785

The largest single-model error group consists of reactions missed only by LocalMapper (1′097 reactions), mainly from superclasses 4, 3, and 9. Among two-model errors, the most frequent pair is the original RXNMapper together with LocalMapper (206 reactions), again enriched in superclasses 4, 9, and 3. In the three-model error class, LocalMapper is the only correct model for 247 reactions, whereas RXNMapper-P is the only correct model for 176 reactions. The 824 reactions missed by all four models present over-represented superclasses 3, 4, and 9. Representative examples from these groups are shown in Fig. 4 and 5, where the difference between ground truth and mistaken predictions are illustrated (picked from the RXNFP TMAP of the error set, Fig. S2).

**Fig. 4:**
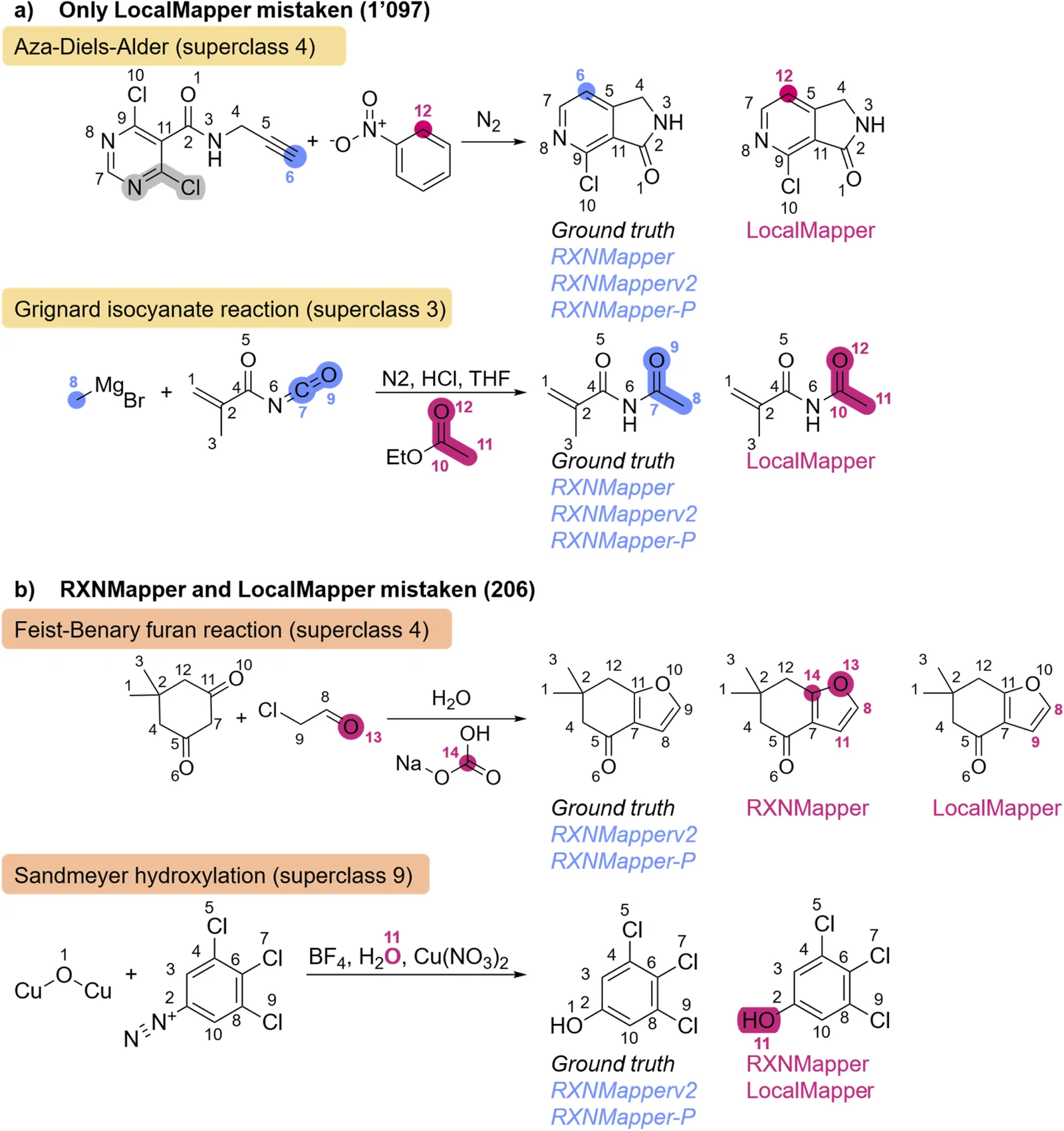
Examples of the 3′785 reactions from the Pistachio test set for which at least one model gives an incorrect prediction. (a) Reactions missed only by LocalMapper.^56^ (b) Reactions missed only by the original RXNMapper and LocalMapper.^57,58^

**Fig. 5:**
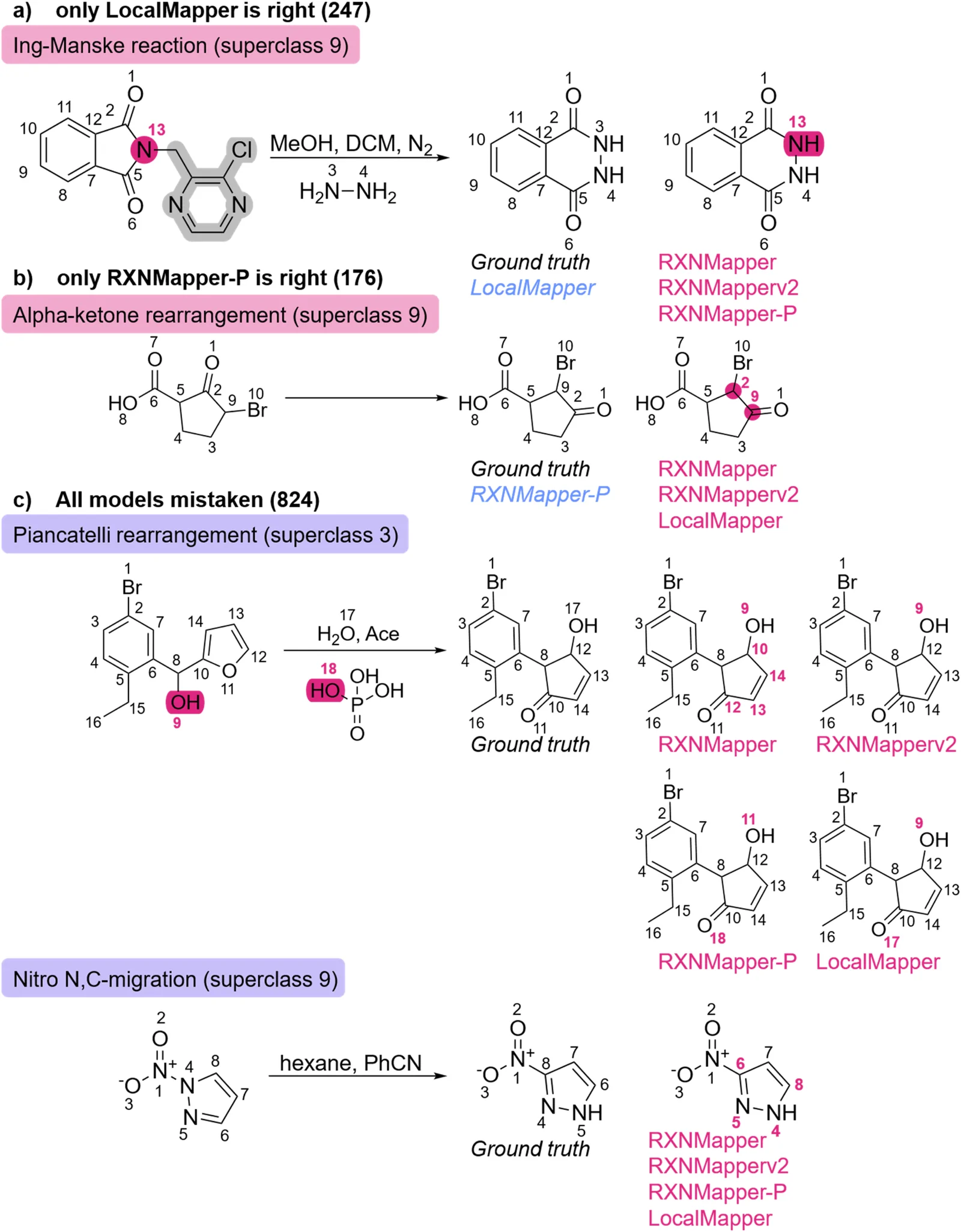
Examples of the 3′785 reactions from the Pistachio test set for which at least one model gives an incorrect prediction. (a) Reaction correctly mapped only by LocalMapper.^59^ (b) Reaction correctly mapped only by RXNMapper-P. (c) Reactions missed by all models.^60,61^

At the superclass level, the models differ mainly in the overall error rate rather than in a clear reaction-type specialization. The two updated RXNMapper models consistently make fewer mistakes than the original RXNMapper and LocalMapper, and RXNMapper-P gives the lowest total error count. The most informative superclasses are 3, 4, and 9 because they combine high frequency with high absolute numbers of errors, whereas superclasses 4 and 8 are the most error-prone relative to class size.

Table 3, and Fig. 4 and 5 show that the largest room for improvement lies in the reactions missed by all models. These 824 cases are less likely to reflect model-specific weaknesses and more likely to correspond to systematic challenges, such as rearrangements, migrations, multi-center transformations, or residual ambiguity in the reference mapping. At the same time, the TMAP does not reveal a clearly separated region of reaction space that is uniquely difficult for one model and easy for another. This suggests that, at the level of NameRXN superclasses, the main difference between models is robustness rather than specialization.

Table 1 demonstrates an increase in atom-mapping performance on average. However, no reaction class drastically improved from always being predicted wrong to only correct predictions. We hypothesize that this comes from the stochastic nature of the training and the lack of direct feedback during the unsupervised MLM training.

Together, Table 2, and Fig. 4 and 5 indicate that future improvements should focus on reaction classes that are both common and error prone. In the present dataset, these are primarily superclasses 3, 4, and 9.

### Mechanistically challenging atom-mapping cases

To move beyond superclass-level trends, we examined representative reaction types that are known or expected to be difficult for atom-mapping algorithms. Fig. 6 shows selected examples from the Pistachio test set together with the alternative mappings predicted by the different models. We focused on rearrangements, for which the atom correspondence is often less intuitive, and on Mitsunobu ester synthesis, which is frequently associated with mapping errors in esterification chemistry.

**Fig. 6:**
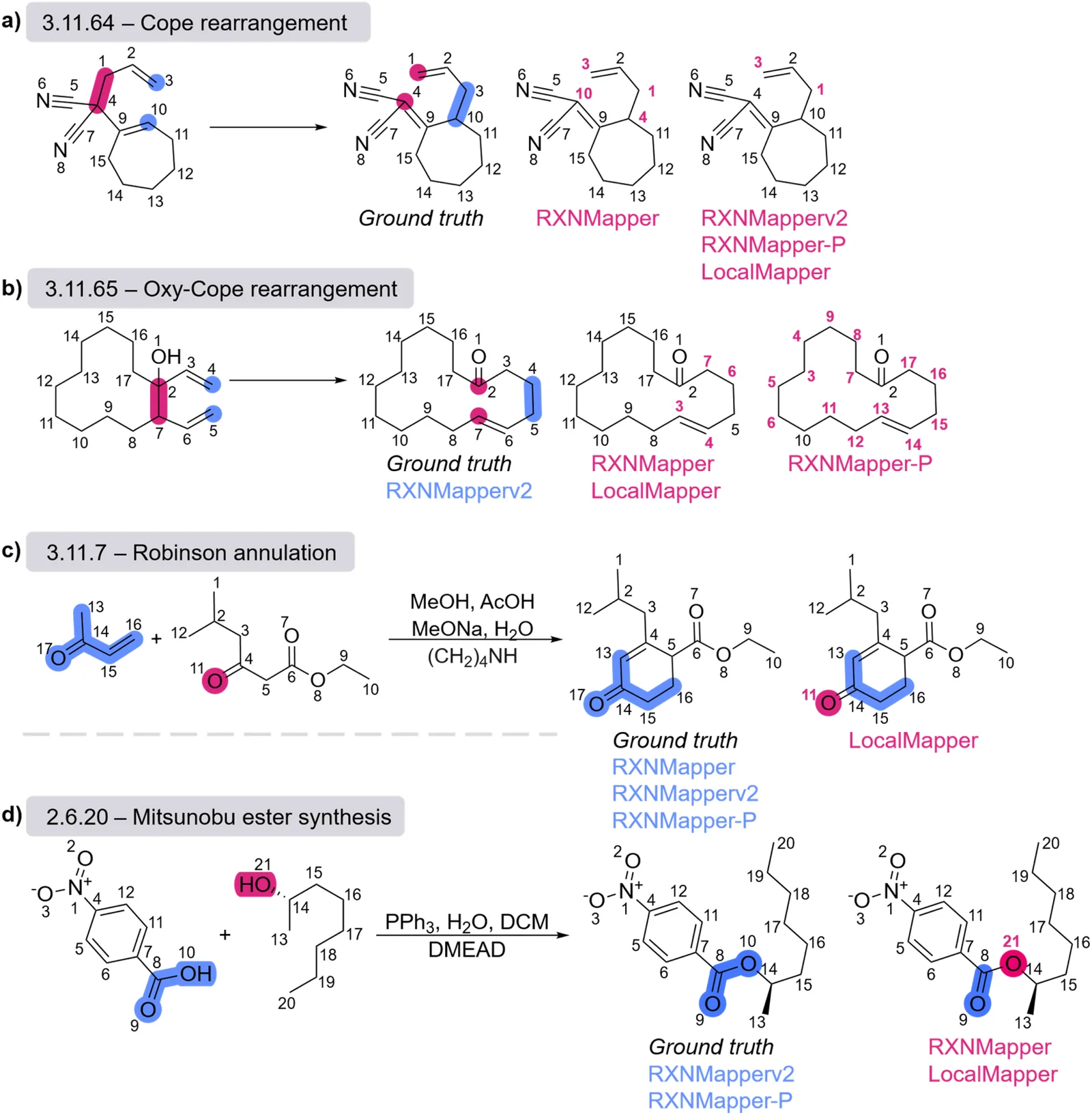
Representative atom-mapping errors on selected difficult reaction types from the Pistachio test set. (a–c) Rearrangement reactions, including Cope^62^ (a), oxy-Cope (b), and Robinson annulation^63^ (c) examples. (d) Mitsunobu ester synthesis.^64^ For each case, the reference mapping and alternative model predictions are shown, highlighting typical failure modes such as missing skeletal rearrangements or incorrect oxygen assignment.

Fig. 6a–c shows representative rearrangements, including Cope, oxy-Cope, and Robinson annulation reactions. Across these examples, the dominant error is failure to capture the underlying skeletal reorganization. Instead, incorrect predictions tend to preserve local structural similarity and assign atom maps as if a substituent had migrated without rearrangement or as if the product double bond arose from the most similar alkene fragment in the reactants. This behavior is particularly apparent for Cope-type transformations, for which most examples are missed by several or all models. Oxy-Cope rearrangements appear somewhat better handled by the updated ALBERT-based models, but the same general failure mode remains. In Robinson annulation, typical errors assign the enone double bond by local similarity rather than by the actual annulation pattern.

Fig. 6d shows a representative Mitsunobu ester synthesis. In this case, the reproduced and Pistachio-trained RXNMapper models recover the reference mapping, whereas the original RXNMapper and LocalMapper assign the ester oxygen incorrectly. The error corresponds to treating the hydroxyl group of the carboxylic acid as the leaving group, a failure mode consistent with other esterification-like transformations in which multiple mechanistic possibilities can compete.

These examples show that the main remaining failures are not random but mechanistically structured. Rearrangements remain difficult because correct atom mapping requires recognition of a global reorganization of the carbon framework rather than simple local similarity matching. Mitsunobu ester synthesis highlights a second source of difficulty, namely competing oxygen assignments in transformations related to ester formation. In both cases, the errors often involve only a small number of exchanged atom labels, yet they correspond to a chemically distinct interpretation of the reaction. This suggests that future improvements will likely require training or evaluation strategies that better account for mechanistic context, especially for rearrangements and esterification-like processes.

## Conclusion

We revisited the RXNMapper approach by addressing two limitations of the original workflow: unreliable validation data and evaluation by direct string matching. Using a curated validation set covering Pistachio chemical space, we retrained ALBERT-based RXNMapper models and optimized head, layer, and checkpoint selection under a common framework. We also developed a preprocessing and graph-based evaluation pipeline that filters ambiguous reference reactions and compares atom mappings at the level of reaction graph equivalence, thereby avoiding false negatives caused by inconsistent formatting or symmetry-equivalent mappings.

Across the golden dataset, the broad Pistachio benchmark, and an internal AstraZeneca medicinal chemistry set, the updated RXNMapper models consistently outperformed the original RXNMapper and LocalMapper. The results show that careful model selection on a representative validation set can improve atom-mapping performance more than retraining on a new reaction corpus alone. At the same time, performance remained strongly dependent on the benchmark, with the broad Pistachio reaction space proving substantially more challenging than the narrower medicinal chemistry set.

Superclass-level analysis and selected case studies further showed that current atom-mapping errors are not uniformly distributed. Frequent and error-prone classes such as C–C bond formation, heterocycle-forming reactions, and functional group interconversions contribute a large fraction of the remaining failures, while rearrangements and Mitsunobu ester synthesis illustrate persistent mechanistic challenges. These cases suggest that future gains will require models and evaluation schemes that better capture mechanistic context rather than local structural similarity alone.

Overall, this work provides both improved RXNMapper models and a more rigorous evaluation framework for atom mapping. We expect that the released training scripts, model-selection workflow, and benchmarking tools will support more reproducible development of atom-mapping algorithms and more reliable evaluation on chemically relevant reaction spaces.

## Author contributions

YG designed and realized the project with SR and wrote the paper. AMW provided technical guidance. SG, PS and JLR designed and supervised the project. JLR wrote the paper. All authors reviewed and approved the final manuscript.

## Conflicts of interest

The authors declare that they have no competing interests.

## Supplementary Material

DD-OLF-D6DD00425C-s001

## Data Availability

The LocalMapper model and inference code are publicly available from the authors' GitHub repository https://github.com/snu-micc/LocalMapper. The updated atom-mapping and training scripts used in this work to retrain ALBERT-based RXNMapper models, the workflow used to select the best head/layer/checkpoint combination, and the evaluation framework for preprocessing and reaction-graph-based comparison of atom-mapping predictions are available at https://github.com/yvsgrndjn/RXNMapper_v2 and https://github.com/yvsgrndjn/atommap_eval. The updated RXNMapper-v2 model checkpoints are available on the GitHub and on HuggingFace (https://huggingface.co/yvsgrndjn/alberta-uspto-2800k). USPTO-full is publicly available and was used here in the version extracted from the United States Patent and Trademark Office reaction corpus by Lowe.^49,50^ USPTO-50 K and the golden dataset are available from their original sources.^3,48^ Pistachio is a proprietary dataset from NextMove Software and is therefore not publicly redistributed. The AstraZeneca medicinal chemistry benchmark used in this study is proprietary and cannot be shared publicly. The updated TMAP code is available on GitHub: https://github.com/afloresep/TMAP2. Supplementary information (SI): Description of reaction graph isomorphism check, overview of Pistachio reaction dataset as TMAPs (Fig. S1 and S2), and examples of incorrect atom mapping predictions (Fig. S3). See DOI: https://doi.org/10.1039/d6dd00425c.
