# Supplementary material for: RXNMapper-v2: updated validation and evaluation on expanded chemical space
Source: Digit Discov. 2026 Oct 13. Online ahead of print. doi: 10.1039/d6dd00425c (PMC13678721; doi:10.1039/d6dd00425c)
Supplement: Supplementary file 1 [file DD-OLF-D6DD00425C-s001.pdf]

Supplementary Information (SI) for Digital Discovery.
This journal is © The Royal Society of Chemistry

Supplementary information - RXNMapperv2: updated validation and evaluation on expanded chemical space

Yves Grandjean,[a] Sacha Raffaud,[b] Annie M. Westerlund,[c] Philippe Schwaller,[b] Samuel Genheden,[c] and Jean-Louis Reymond[a]*

[a)] *Department of Chemistry, Biochemistry and Pharmaceutical Sciences, University of Bern, Freiestrasse 3, 3012 Bern, Switzerland*

[b)] *Ecole Polytechnique Fédérale de Lausanne (EPFL), Lausanne, Switzerland.*

[c)] *Molecular AI, Discovery Sciences, R&D, AstraZeneca Gothenburg, SE-431 83 Mölndal, Sweden*

e-mail: *jean-louis.reymond@unibe.ch*

Theory

Reaction graph isomorphism check. Following the work from Laffitte *et al.*[56] on comparison of atom mappings from a mathematical perspective, we build reaction graphs for both the ground truth and the predicted atom-mapped reactions. Both reactions are considered chemically equivalent if the reaction graphs are isomorphic as colored graphs. More formally, let M be a molecule appearing on the reactant or product side. The package converts M into an undirected labeled graph:

$$G(M) = G(V(M), E(M), \mathrm{l}_V, \mathrm{l}_E)$$

Each heavy atom, a_i, of M corresponds to a vertex in the graph: $V(M) = \{a_1, \dots, a_n\}$, while each covalent bond, $b_k = \{a_i, a_j\}$, of M corresponds to an undirected edge: $E(M) \subseteq \{\{a_i, a_j\}: a_i, a_j \in V(M), i \neq j\}$.

For each atom $a_i \in V(M)$, the implemented node attributes are $\mathrm{l}_V(a_i) = \big(Z(a_i), \mu(a_i), q(a_i), d(a_i)\big)$, where $Z(a_i)$ is the atomic number, $\mu(a_i)$ is the atom-map number, $q(a_i)$ is the formal charge and $d(a_i)$ is the atom degree.

For each bond $b_k = \{a_i, a_j\} \in E(M)$, the implemented edge label is $\mathrm{l}_E(b_k) = \tau(b_k)$ where $\tau(b_k)$ is the bond type as encoded by RDKit and converted to a string.

If a molecule contains no mapped atom, then it is discarded before building the reaction graph. In other words if

$$\max_{a_i \in V(M)} \mu(a_i) = 0,$$

then M is removed from the construction. Hence the reaction graph is built only from molecules containing at least one atom a such that $\mu(a) > 0$.

Let

$$M_R = \big(M_{R,1}, \dots, M_{R,n_R}\big),$$

$$M_P = \big(M_{P,1}, \dots, M_{P,n_P}\big)$$

be the lists of retained mapped reactant and product molecules, respectively. For each retained molecule, we build its molecular graph:

$$G_{R,i} = G(M_{R,i}), \qquad G_{P,j} = G(M_{P,j}).$$

We then form the disjoint unions

$$G_R = \bigsqcup_{i=1}^{n_R} G_{R,i}\,,$$

$$G_P = \bigsqcup_{j=1}^{n_P} G_{P,j}.$$

and define the union graph as $G_{\cup} = G_R \sqcup G_P$.

Finally, the reaction graph is obtained by adding special edges between reactant and product atoms that share the same (strictly positive) atom-map number.

For each $k \in \mathbb{N}_{>\mathbb{0}}$, define

$$R_k = \{a_i \in V(G_R) \colon \mu(a_i) = k\},$$

$$P_k = \{a_j \in V(G_P) \colon \mu(a_j) = k\}.$$

The algorithm adds all edges

$$\{a_i, a_j\} \quad \text{for all } a_i \in R_k,\ a_j \in P_k.$$

Each such edge is labeled by a dedicated marker, denoted here by

$$\mathrm{l}_E(\{a, b\}) = mapping.$$

Therefore the final reaction graph of reaction ρ is

$$G_{\mathrm{rxn}}(\rho) = \left(V_\cup, E_\cup \cup E_{\mathrm{map}}, \mathrm{l}_V, \mathrm{l}_E\right),$$

where $E_{\mathrm{map}} = \bigcup_{k>0}\left\{\{a_i, a_j\}: a_i \in R_k,\ a_j \in P_k\right\}$ contains all edges in the reaction graph between product and reactant atoms with identical atom-map number. Under an ideal atom mapping, each map number appears at most once on the reactant side and at most once on the product side, so each k contributes at most one mapping edge. Our framework allows for multiple occurrences and will create a complete bipartite connection between all same-labeled reactant and product atoms if repeated map numbers occur.

Given two atom-mapped reactions ρ_1 and ρ_2, build their reaction graphs

$$G_1 = G_{\mathrm{rxn}}(\rho_1),$$

$$G_2 = G_{\mathrm{rxn}}(\rho_2).$$

The package declares the two atom mappings equivalent if and only if G_1 and G_2 are isomorphic as labeled graphs with the following matching rules.

Nodes must agree on:

$$\left(Z(a), q(a), d(a)\right).$$

Notably, the atom-map number $\mu(a)$ is not matched directly, equivalence must be invariant under a global renaming of atom-map indices.

Edges must agree on:

- bond type for intramolecular bond edges,
- the special label `mapping` for reactant-product mapping edges.

Thus the comparison is a colored graph isomorphism test:

$$G_1 \cong G_2.$$

Equivalently, there must exist a bijection

$$\varphi: V(G_1) \rightarrow V(G_2)$$

such that:

1. φ preserves adjacency,

2. φ preserves bond-edge labels,

3. φ preserves mapping-edge labels,

4. φ preserves the node labels (Z, q, d).

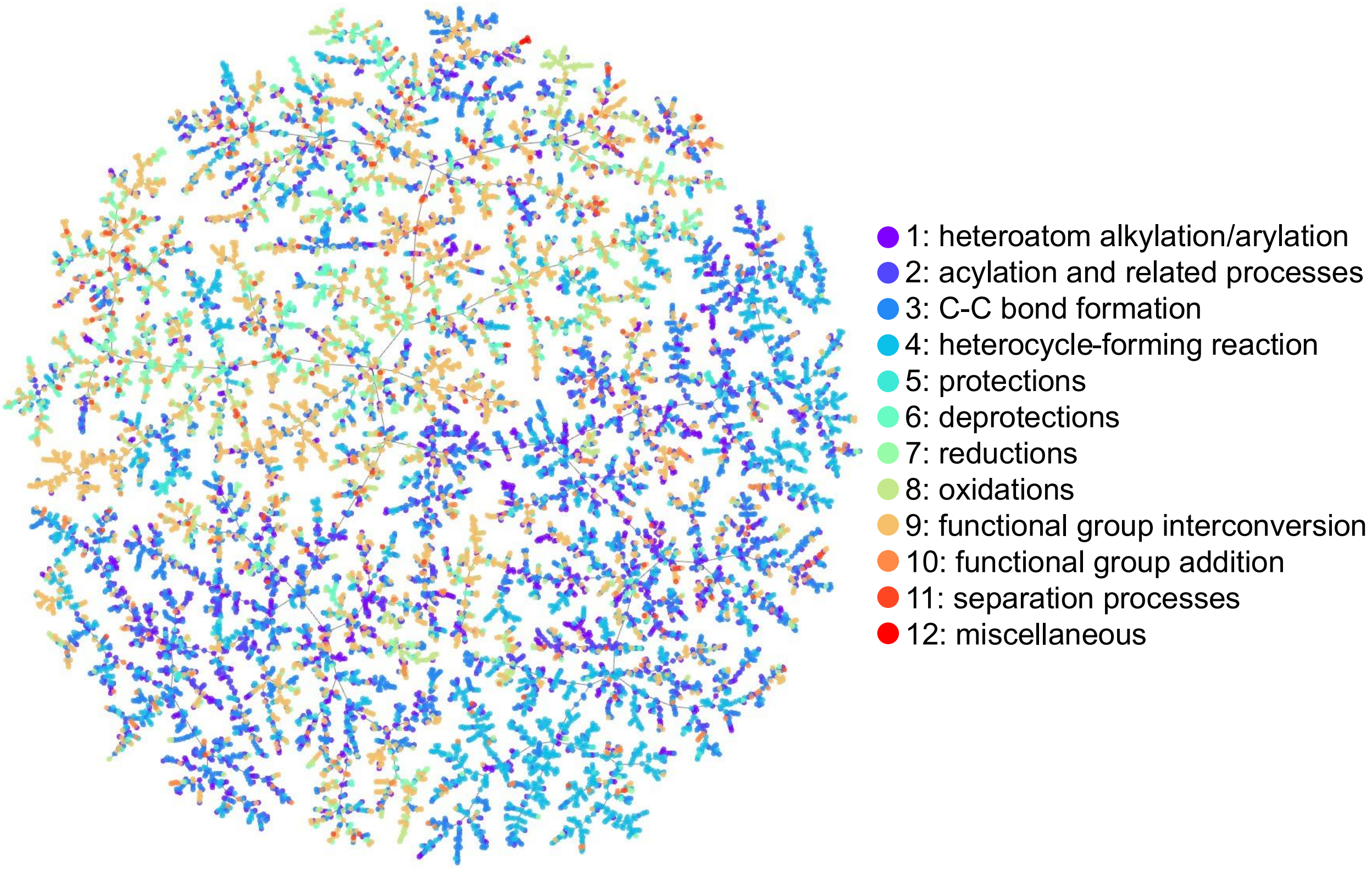

Figure S1: RXNFP TMAP of the 13'862 reactions from the Pistachio test set, colored by NameRXN superclass.

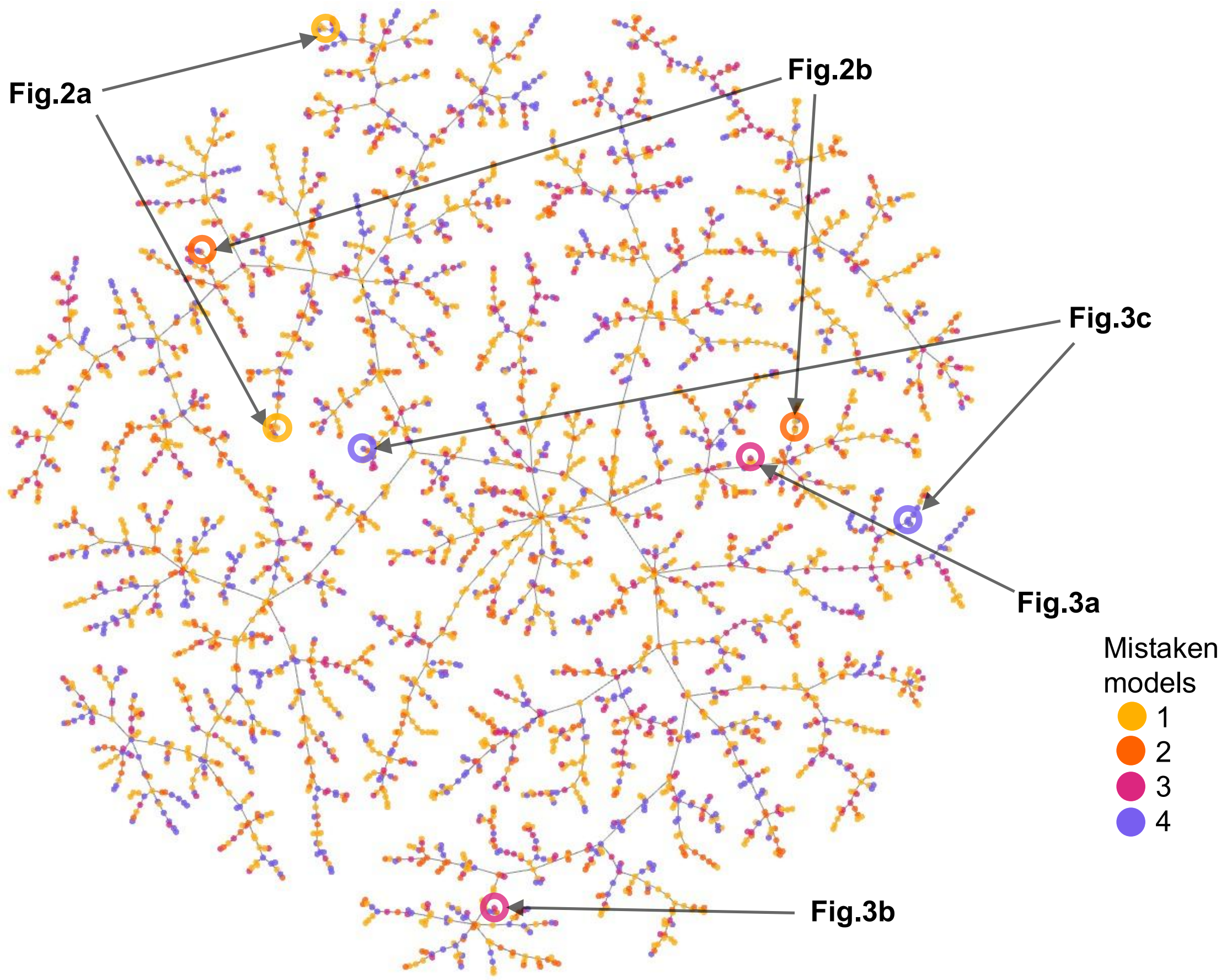

Figure S2: RXNFP TMAP of the 3'785 reactions from the Pistachio test set that were predicted wrong by at least one model. The reactions pointed by arrows are the ones shown in **Figures 2,3**.

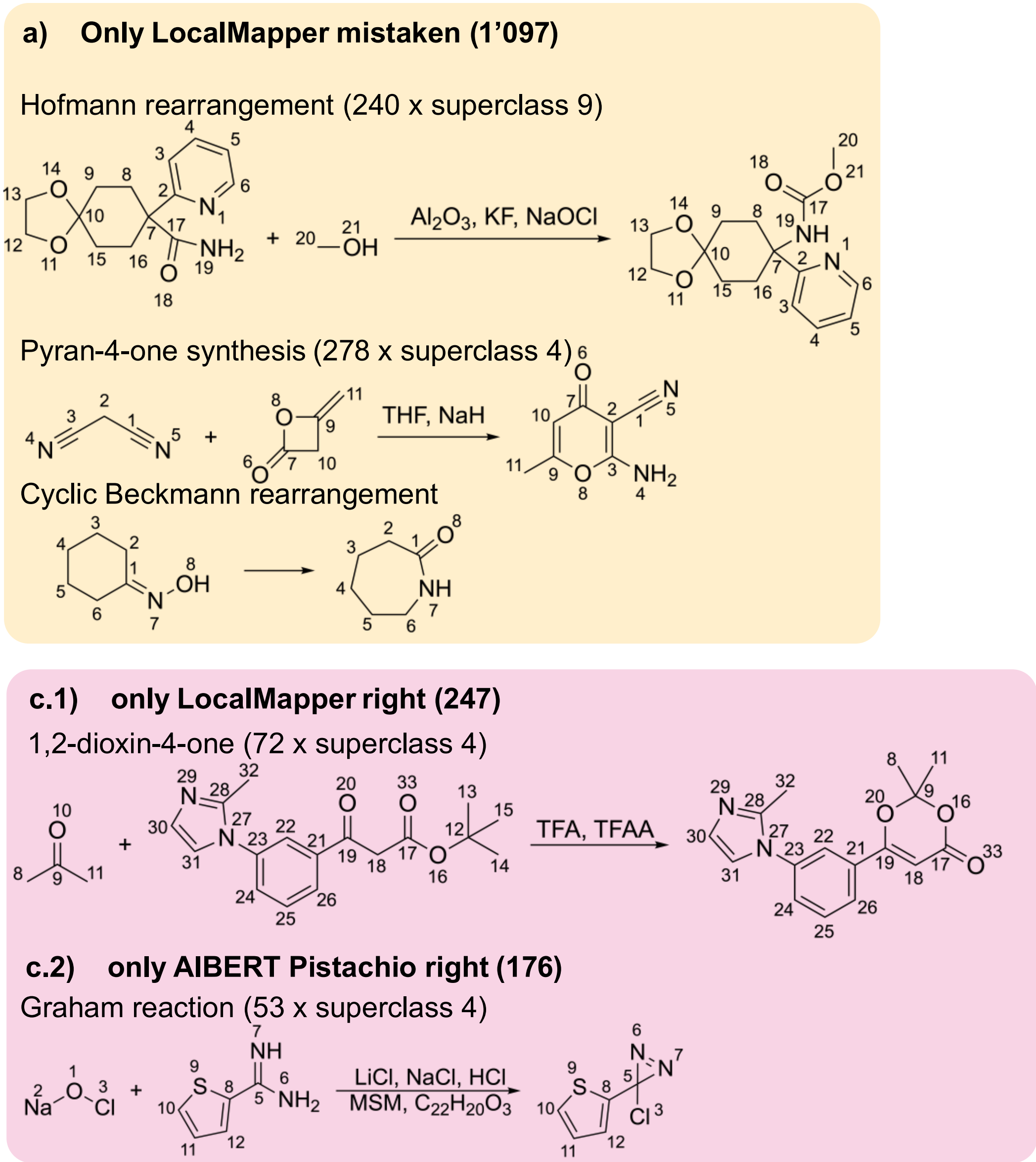

Figure S3: Part 2, RXNFP TMAP of the 3'785 reactions from the Pistachio test set for which at least once model gives an incorrect prediction. **a)** Reactions missed only by LocalMapper. **b)** None shown **c.1)** Reaction correctly mapped only by LocalMapper. **c.2)** Reaction correctly mapped only by RXNMapper-P. **d)** None shown.
